# Malaria hotspots defined by clinical malaria, asymptomatic carriage, PCR and vector numbers in a low transmission area on the Kenyan Coast

**DOI:** 10.1186/s12936-016-1260-3

**Published:** 2016-04-14

**Authors:** David Tiga Kangoye, Abdisalan Noor, Janet Midega, Joyce Mwongeli, Dora Mkabili, Polycarp Mogeni, Christine Kerubo, Pauline Akoo, Joseph Mwangangi, Chris Drakeley, Kevin Marsh, Philip Bejon, Patricia Njuguna

**Affiliations:** Kenya Medical Research Institute-Wellcome Trust Research Programme, Centre for Geographic Medicine Research, P.O. Box 230, Kilifi, 80108 Kenya; Department of Infectious and Tropical Diseases, London School of Hygiene and Tropical Medicine, London, UK; Nuffield Department of Medicine, Centre for Clinical Vaccinology and Tropical Medicine, Churchill Hospital, University of Oxford, Oxford, UK

**Keywords:** Malaria, Hotspots, Spatial scan statistic, Antibodies, Serology, Asymptomatic parasitemia, Transmission, Targeted intervention

## Abstract

**Background:**

Targeted malaria control interventions are expected to be cost-effective. Clinical, parasitological and serological markers of malaria transmission have been used to detect malaria transmission hotspots, but few studies have examined the relationship between the different potential markers in low transmission areas. The present study reports on the relationships between clinical, parasitological, serological and entomological markers of malaria transmission in an area of low transmission intensity in Coastal Kenya.

**Methods:**

Longitudinal data collected from 831 children aged 5–17 months, cross-sectional survey data from 800 older children and adults, and entomological survey data collected in Ganze on the Kenyan Coast were used in the present study. The spatial scan statistic test used to detect malaria transmission hotspots was based on incidence of clinical malaria episodes, prevalence of asymptomatic asexual parasites carriage detected by microscopy and polymerase chain reaction (PCR), seroprevalence of antibodies to two *Plasmodium falciparum* merozoite antigens (AMA1 and MSP1-19) and densities of *Anopheles* mosquitoes in CDC light-trap catches.

**Results:**

There was considerable overlapping of hotspots by these different markers, but only weak to moderate correlation between parasitological and serological markers. PCR prevalence and seroprevalence of antibodies to AMA1 or MSP1-19 appeared to be more sensitive markers of hotspots at very low transmission intensity.

**Conclusion:**

These findings may support the choice of either serology or PCR as markers in the detection of malaria transmission hotspots for targeted interventions.

**Electronic supplementary material:**

The online version of this article (doi:10.1186/s12936-016-1260-3) contains supplementary material, which is available to authorized users.

## Background

“Malaria hotspots” are defined as geographical areas within a wider area of transmission in which the transmission intensity is significantly higher than the average level in the surrounding area of that setting and are widely observed in malaria endemic regions [1]. Stable and unstable hotspots have been reported in Kilifi [2] and they occur at various scales ranging from regional to homestead level [3].

Factors that are likely to determine the risk and spread of malaria include environmental factors such as temperature, altitude, distance to water bodies, wind direction, and urbanization [4–6]. They also include intrinsic human characteristics, such as red blood cell genetic polymorphisms, differential host attractiveness to *Anopheles* mosquitoes, fetal haemoglobin and dietary factors in early infancy, and extrinsic factors such as agricultural practices, socio-economic factors, housing, level of education and behaviour [7–15].

Hotspots represent an opportunity for targeted control interventions that are expected to be more efficient than untargeted interventions and ultimately benefit the whole community [16].

Challenges in the identification of hotspots of transmission include the choice of the transmission marker to measure, the choice of the geospatial method of detection, the choice of the scale of detection, when to detect them and how stable they are [16, 17]. Asymptomatic parasite carriage, clinical malaria episodes, vector biting intensities or antibody responses to selected malaria antigens have been proposed as potential markers of malaria transmission in detecting hotspots in areas of low to moderate transmission intensity [18]. The exploration of PCR and serology as transmission markers has been especially suggested in areas of unstable or very low transmission intensity [19, 20].

In the present study, simultaneous measurements of several malaria transmission indicators were carried out in an area of low transmission including clinical, parasitological, serological and entomological markers. These different markers were then used to detect malaria transmission hotspots, to examine the spatial overlapping of the specific hotspots and to analyse the correlations between the markers. Ultimately, this study aims at providing additional evidence that might guide the choice of markers to be used in the detection of malaria transmission hotspots.

## Methods

### Ethical approval

The Kenya Medical Research Institute (KEMRI) Ethical Review Committee approved the Mal055 study (SSC 1445) and the MTI study (SSC 2072). Study procedures were explained, and written informed consent was sought and obtained from each participant or his parents/guardians (for children) prior to any study procedure. The study was conducted according to the Declaration of Helsinki.

### Study area, population and surveillance method

The data used in the present study were taken from studies in Kilifi county on the Kenyan Coast. There were two cohorts monitored, one for clinical episodes during 2 years of follow up and a second cohort monitored via cross-sectional surveys. The data were collected from January 2012 to December 2013 for the longitudinal monitoring and from July to September 2012 and May to July 2013 for the first and second cross-sectional surveys respectively.

831 children aged 5–17 months residing in 633 homesteads were recruited into a randomized, controlled malaria vaccine trial in which longitudinal monitoring of malaria episodes was done [21]. Febrile malaria episodes were detected by passive case detection as previously described [21]. Clinical malaria was defined as the presence of fever (axillary temperature ≥37.5 °C) or history of fever in the past 24 h and parasitaemia ≥2500/μL [22].

In the same study area, two cross-sectional surveys involving 800 individuals (children and adults) were conducted in 2012 and 2013. The distribution of this population by age group is shown in Additional file 1. The homesteads, in which these participants were residing (211 and 183 in 2012 and 2013 respectively), were selected among 2456 homesteads recorded in sub-locations of the study administrative area by simple random sampling. The households involved in the malaria vaccine study were excluded. The cross-sectional surveys were used to measure asymptomatic parasitaemia, by microscopy of thick and thin blood smears and by PCR as described elsewhere [23], and IgG specific antibody responses to *Plasmodium falciparum* merozoite antigens [the apical membrane antigen 1 (AMA1) and the 19 kDa C-terminal region of the merozoite surface protein 1 (MSP1-19)] as previously described [24]. Travel data were not recorded for any of these studies.

**Fig. 1:**
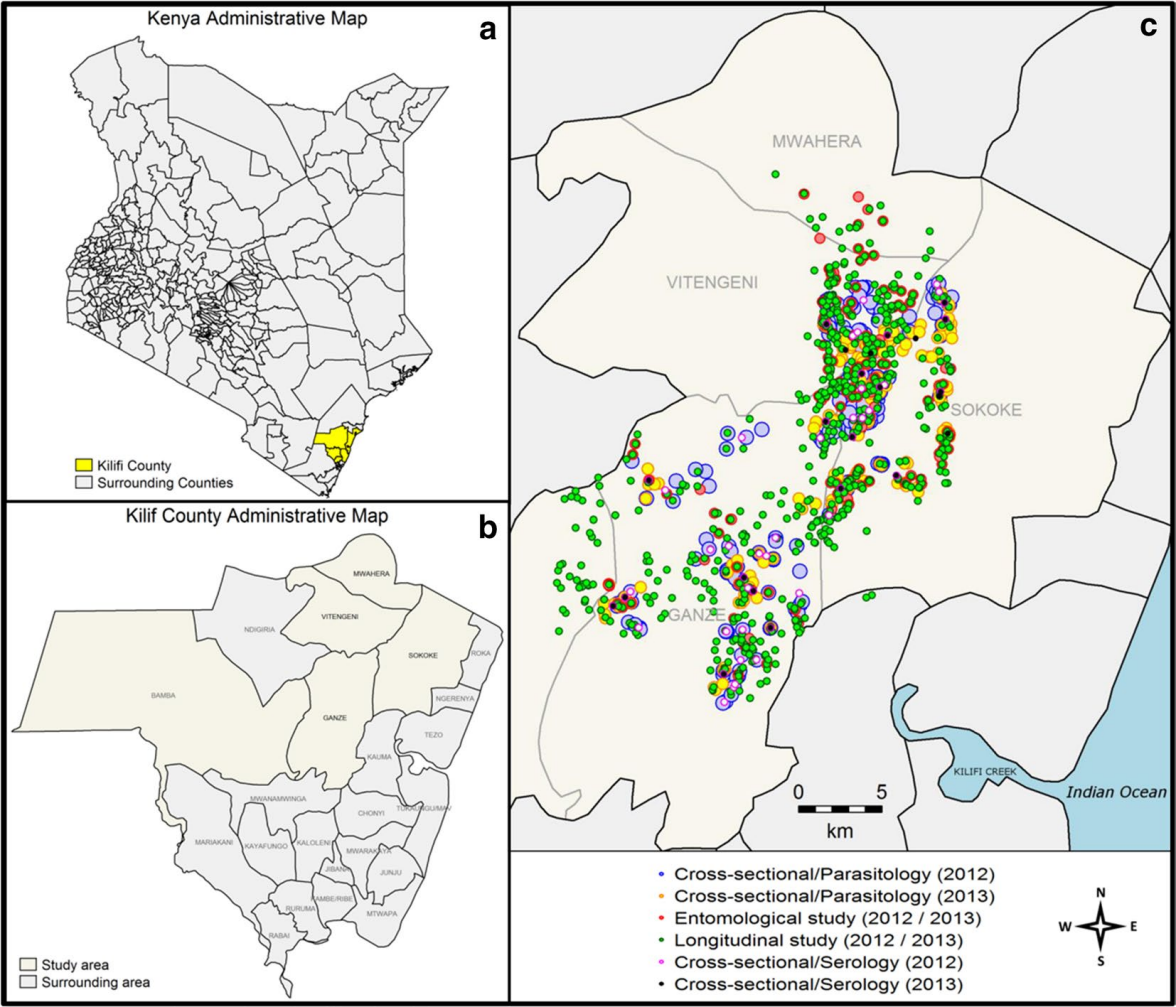
Study area. *Panels* **a** and **b** show respectively a map of Kenya with a focus on Kilifi County and a map of Kilifi County highlighting the study area. On *panel* **c**, each *dot* represents the location of a homestead. The *colors* uniquely identify the studies. The different sizes of the *dots* help distinguish very close homesteads

Data from an entomological study conducted in the same study area were also available for 2012 and 2013. Mosquito captures using CDC light traps were conducted in 150 sampled houses, chosen at random, at six rounds covering the dry season, the long and the short rains. *Anopheles gambiae* and *Anopheles funestus* were the major human malaria vectors captured in the study area. The homesteads involved in the entomological study were not the same as those sampled for the clinical surveillance of children, and this limited the possibility to examine the association between household level mosquito exposure and household level malaria incidence. Longitude and latitude data for each homestead involved in the clinical and entomological studies were recorded during the surveys using handheld Garmin eTrex Global Positioning System devices. The study area, along with the distribution of the different homesteads involved, is illustrated in Fig. 1.

### Detection of hotspots

Using the scan statistic method by Kulldorff [25], clusters of significantly higher risk of malaria than the remaining surrounding study area were detected using clinical, biological and entomological markers. The following markers were examined: clinical malaria, positive blood films, positive PCR tests, seropositivity to AMA1 and MSP1-19 and densities of *Anopheles* mosquitoes. The cut offs for seropositivity to AMA1 and MSP1-19 in normalized optical density were, respectively 0.132 and 0.108 in 2012, and 0.091 and 0.13 in 2013; they were determined using previously described methods [26]. The application of the scan statistic by SaTScan has been described previously [25]. Briefly, a scanning window (set to “circular” in the present analysis) is moved across the study area, and the maximum number of events that are captured by the window is recorded. The maximum window size was set to 30 % of the population at risk. Each of the different scanning windows was evaluated as a potential cluster by the calculation of a likelihood ratio test statistic based on the observed, expected and total number of cases. The corresponding p value is calculated using a Monte Carlo method.

To detect hotspots of clinical malaria cases, we used a discrete Poisson model where the cases were the clinical malaria cases detected in each homestead; population was defined as all monitored individuals residing in the corresponding homesteads. A Bernoulli probability model was used to detect hotspots of positive blood films, hotspots of positive PCR tests and hotspots of individuals seropositive for AMA1 and MSP1-19. Cases were defined as individuals with a positive test (blood film, PCR or ELISA) in each homestead; the controls were defined as the individuals with negative tests in the corresponding homesteads. A discrete Poisson model, in which the cases were the *Anopheles* mosquitoes captured in each house, was used to detect hotspots of *Anopheles* mosquitoes; the population was defined as one individual per house.

For each detected hotspot, a relative risk (RR) was computed. The RR is the magnitude of the risk of malaria for individuals residing within the hotspot compared with those residing outside the hotspot. It is calculated as the ratio of the estimated risk within the hotspot and the estimated risk in the surrounding area. The estimated risk is calculated as the number of observed cases divided by the number of expected cases if the null hypothesis was true i.e. if the distribution of cases was totally random. The threshold for statistical significance of the hotspots was set to 0.05.

Two hotspots overlap when the distance between their centres is smaller than the sum of their radii. The degree of overlapping of hotspots was estimated by calculating, for a given couple of hotspots, the ratio of the common surface area by the total surface area of the smallest hotspot. The common surface area (A) of the intersecting hotspots is given by the following formula [27]:

$$\begin{aligned} A =&\, r^{2 } \cos^{ - 1} \left(\frac{{d^{2} + r^{2} - R^{2} }}{2dr}\right) + R^{2} \cos^{ - 1} \left(\frac{{d^{2} - r^{2} + R^{2} }}{2dR}\right) \\ &\quad - \frac{1}{2}\sqrt {\left( { - d = r + R} \right)\left( {d + r - R} \right)\left( {d - r + R} \right)\left( {d + r + R} \right)} \end{aligned}$$

where *R* and *r* are the radii of the hotspots and *d* the distance between their centres. When the smallest hotspot is entirely covered by the bigger hotspot with no intersection or when two concentric hotspots exactly overlap, a ratio = 1 is assigned.

Observations with missing coordinates data were 4.5, 3 and <1 % for serological, entomological and clinical surveillance data respectively; they were dropped prior to any analysis. Missing data for *Anopheles* mosquito capture and AMA1/MSP1-19 serology were <1 % in the respective datasets.

### Statistical analysis

To examine the distribution of the markers of malaria transmission, the data were summarized at homestead level by calculating sum of clinical malaria cases, sum of positive blood films, sum of positive PCR tests and geometric mean antibody titre.

The data were then aggregated using a raster map of the study area derived from a Kilifi county administrative map (see Additional file 2); the original shapefile was downloaded from [28]. The resolution of the raster surface was set to 0.9 km. At this resolution and for each marker, each homestead was assigned to a unique cell by computing the shortest distance between the index homestead and the surrounding grid points. The values of each of the markers at homestead level were then aggregated at grid cell level. The statistics used to aggregate the markers were the mean for counts of positive blood films and positive PCR tests, the weighted mean for count of clinical malaria cases and the weighted geometric mean for antibody titres. This aggregation was repeated for each year. The spatial correlations between these markers were examined using Spearman's rank correlation coefficient on the aggregated data. SaTScan™ v9.4.1 was used to detect the hotspots and Stata 13.1 for Windows, StataCorp LP was used to perform the data analysis and produce the maps.

## Results

### Malaria morbidity and transmission indicators in the study area

The clinical, parasitological, serological and entomological markers measured in these studies are summarized in Table 1. Malaria transmission intensity was low in 2012 with parasite rate by microscopy at 2 % in the general population, and declined further to 0.2 % in 2013. Similarly, there was a decline in densities of malaria vectors in the study area in 2013 with *Anopheles* mosquitoes captured in only 6 % of the surveyed houses compared with 24 % in 2012.

**Table 1:** Yearly summary of clinical, parasitological, serological and entomological markers in the study area

	Year	2012	2013
Longitudinal study	Homesteads/population	633/831	633/831
	Homesteads with malaria	65 (10.3 %)	22 (3.5 %)
	Malaria cases	112 (13.5 %)	28 (3.4 %)
Cross-sectional studies	Homesteads/population	211/779	183/797
	Homesteads with positive blood films	8 (3.8 %)	2 (1.1 %)
	Homesteads with positive PCR tests	31 (14.7 %)	22 (12 %)
	Prevalence asymptomatic infection (microscopy), (95 % CI)	2 % (1.2–3.2)	0.2 % (0.03–0.9)
	Prevalence asymptomatic infection (PCR), (95 % CI)	6.2 % (4.6–8)	3.3 % (2.2–4.8)
	Seroprevalence of antibodies to AMA1, (95 % CI)	36.1 % (32.7–39.5)	20.4 % (17.7–23.4)
	Seroprevalence of antibodies to MSP1-19, (95 % CI)	19.9 % (17.1–22.9)	10.5 % (8.5–12.9)
Entomological surveys	Homesteads	145	142
	Homesteads with *Anopheles*	35 (24 %)	8 (6 %)
	Range of *Anopheles* captured/house	0–17	0–6
	Total *Anopheles* captured	101	15
	Total *An. gambiae* captured	85	5
	Total *An. funestus* captured	16	10

### Malaria hotspots in the study area

**Fig. 2:**
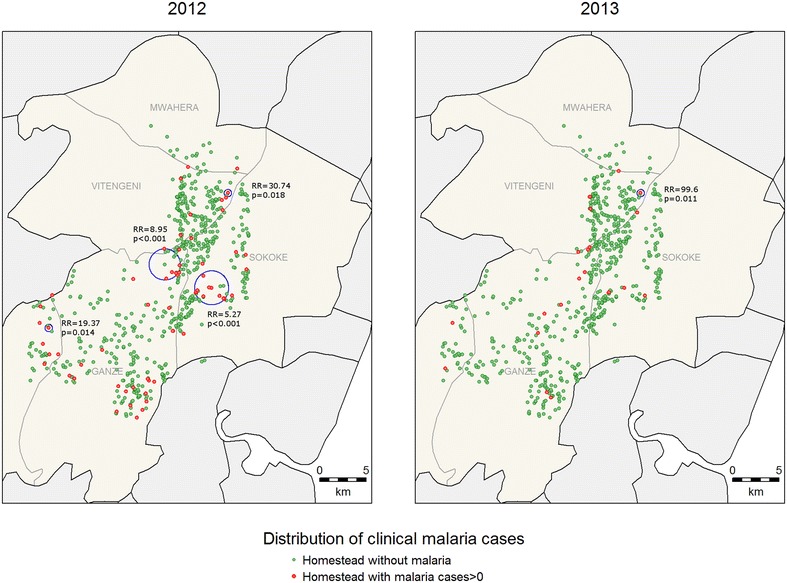
Hotspots of clinical malaria cases. Each *blue circle* represents a statistically significant hotspot with its relative risk (RR) and p value displayed beside the circle

**Fig. 3:**
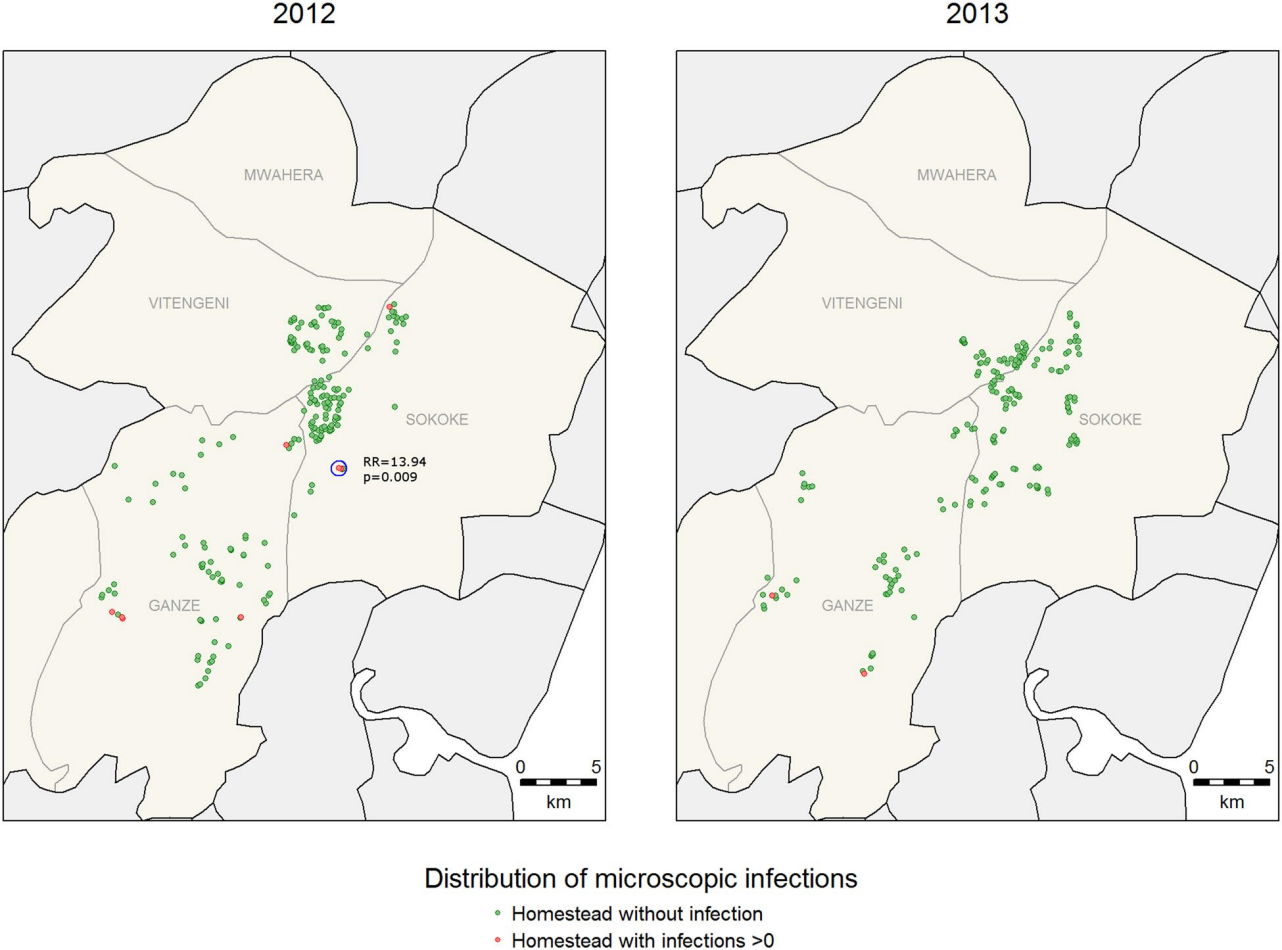
Hotspots of asymptomatic malaria infections detected by light microscopy. Each *blue circle* represents a statistically significant hotspot with its relative risk (RR) and p value displayed beside the circle. No hotspot was identified in 2013

**Fig. 4:**
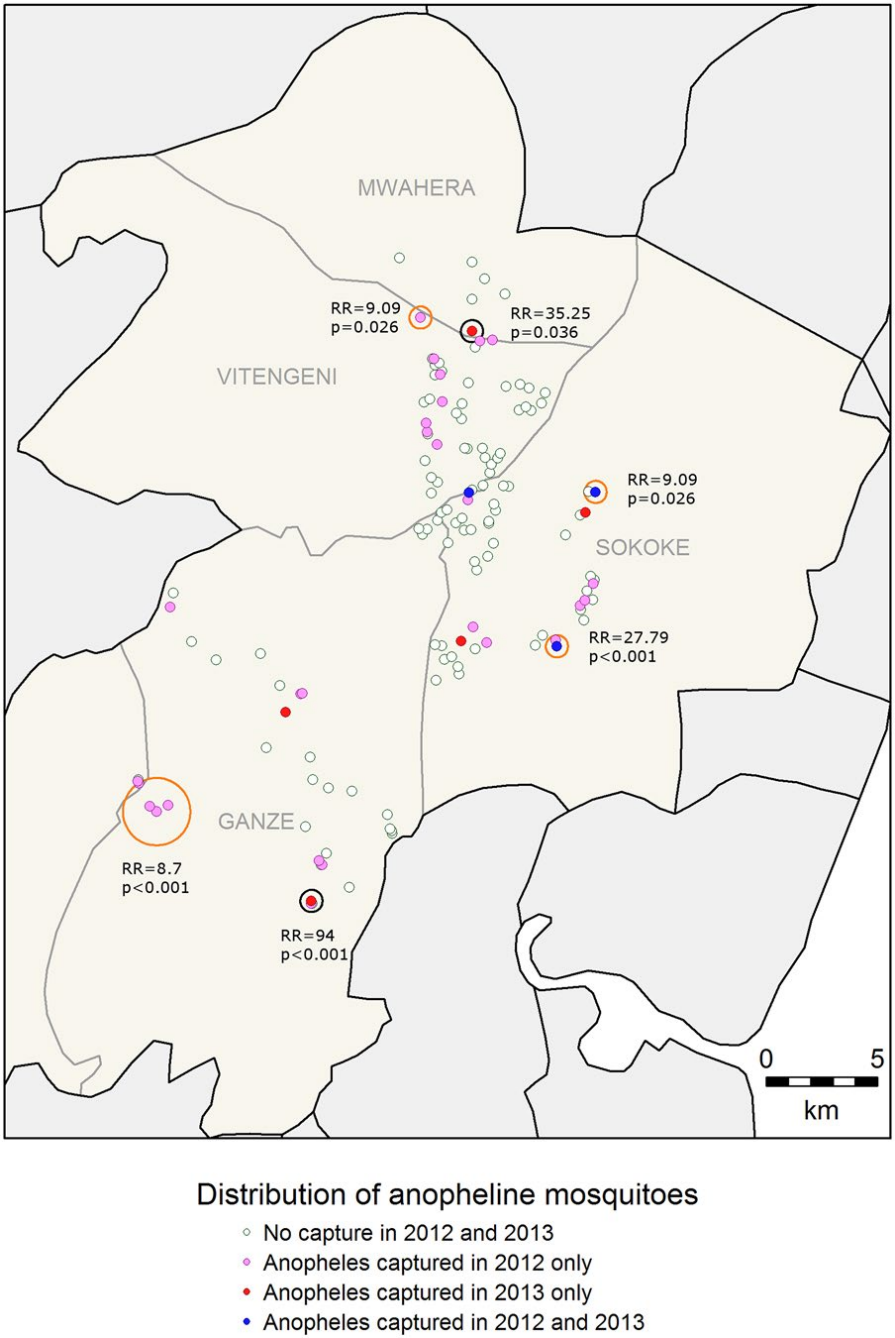
Hotspots of *Anopheles* mosquitoes*. Anopheles gambiae* and *Anopheles funestus* were the only human malaria vector species captured during the survey. The *orange* and *black circles* represent the statistically significant hotspots of *Anopheles* mosquitoes in 2012 and 2013 respectively. Each hotspot is displayed with its malaria relative risk (RR) and p value

**Fig. 5:**
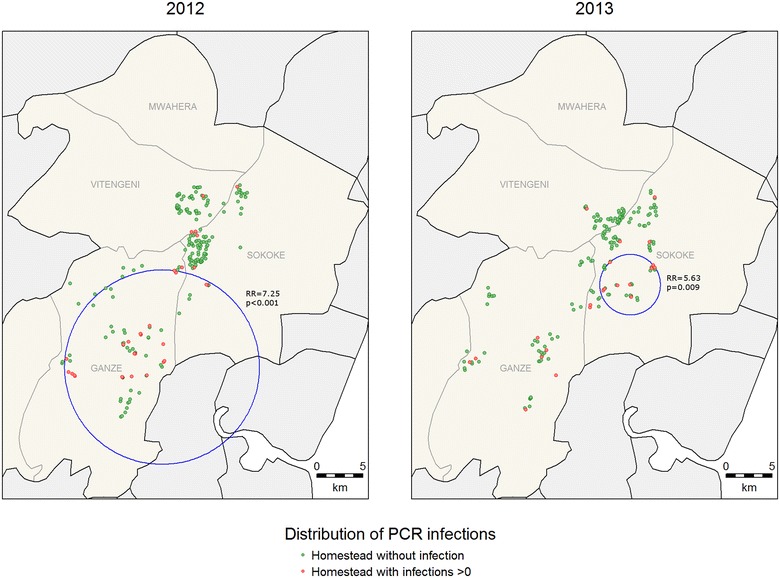
Hotspots of asymptomatic malaria infections detected by polymerase chain reaction (PCR). Each *blue circle* represents a statistically significant hotspot with its relative risk (RR) and p value displayed beside the circle

**Fig. 6:**
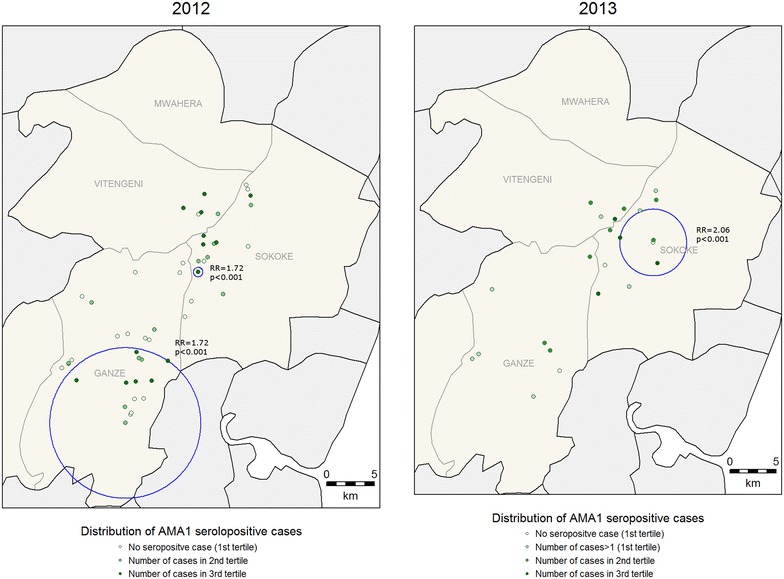
Hotspots of individuals seropositive to *Plasmodium falciparum* apical membrane antigen 1 (AMA1). Each *blue circle* represents a statistically significant hotspot with its relative risk (RR) and p value displayed beside the circle

**Fig. 7:**
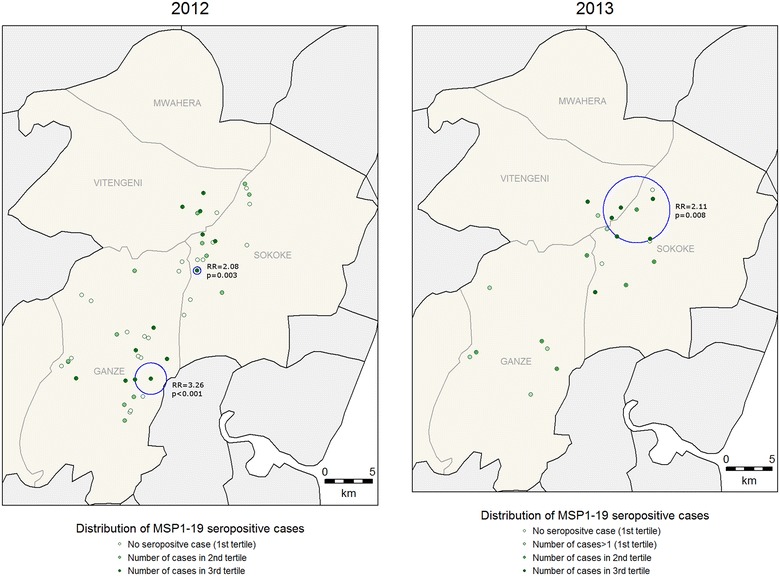
Hotspots of individuals seropositive to *Plasmodium falciparum* merozoite surface protein 1 (MSP1-19). Each *blue circle* represents a statistically significant hotspot with its relative risk (RR) and p value displayed beside the circle

For clinical malaria, asymptomatic parasitaemia determined by microscopy and *Anopheles* mosquitoes captures, there were fewer positive cases in 2013 compared to 2012 (Table 1) and fewer hotspots were identified (Figs. 2, 3, 4). In 2013, the PCR and AMA1, but not MSP1-19, hotspots were reduced to 10 % and 21 % of their size in 2012 respectively (Figs. 5, 6, 7); the clinical malaria and mosquito vector hotspots were limited to single homesteads (Figs. 2, 4). There was one stable hotspot of clinical malaria (Fig. 2) in terms of position and size, limited to a single homestead, but the three other hotspots identified in 2012 were not identified in 2013. No hotspots of asymptomatic parasitaemia detected by microscopy was observed in 2013 (Fig. 3).

The sampling of homesteads for the cross-sectional surveys differed between 2012 and 2013 (Fig. 1). However the hotspot of asymptomatic parasitaemia detected by PCR in 2012 overlapped 39 % of the one identified in 2013 (see Additional files 3 and 4). Moreover, globally, the hotspots of children seropositive to AMA1 overlapped 100 % and 38 % of the hotspots of children seropositive to MSP1-19 in 2012 and 2013 respectively. When the marker used was seropositivity to both AMA1 and MSP1-19, the hotspots detected in 2012 globally overlapped 100 % and 4.4 % of the hotspots of individuals seropositive to MSP1-19 and AMA1 respectively. In 2013, the pattern was reversed with the hotspot of children seropositive to both AMA1 and MSP1-19 overlapping 100 % and 57 % of hotspots of children seropositive to AMA1 and MSP1-19 respectively (see Additional file 5). There was one location in 2012 where the three types of serology hotspots (i.e. seropositive to AMA1, seropositive to MSP1-19 and seropositive to both AMA1 and MSP1-19) exactly overlapped each other (see Additional file 5).

Variable overlapping of hotspots of clinical and parasitological markers of transmission occurred in 2012 (see Additional files 6 and 7). Two hotspots of clinical malaria and two hotspots of *Anopheles* mosquitoes, limited to single-homesteads, did not overlap the hotspot determined by positive PCR tests. The hotspot of asymptomatic parasitaemia detected by microscopy was totally contained within the hotspot determined by positive PCR tests.

Although the data were collected from different samples of homesteads for the different markers, these homesteads were all contained within the same single study area. The different hotspots were visualized together to further investigate the extent of intersection and overlapping between them (see Additional file 8). Most hotspots concentrated in the southern part of the study area in 2012 and moved towards the northern part in 2013. Seventy-one and sixty-six percent of hotspots overlapped with each other in 2012 and 2013 respectively, irrespective of the markers. Considerable overlapping was observed between the different hotspots as detailed in Additional files 7 and 9. The substantial overlapping of the AMA1 hotspot with that of asymptomatic infections detected by PCR observed in 2012 was also observed in 2013 when the transmission declined further.

### Spatial correlations between markers of malaria transmission

The distribution of the different markers is shown in Additional files 10 and 11. With the exception of *Anopheles* mosquito densities, weak to moderate statistically significant correlations were found between the other markers of transmission in 2012 when the markers were averaged at grid cell level (see Table 2 and Additional file 12). Clinical malaria was weakly correlated with positive blood films (r^2^ = 0.07) but not with any other marker. Serological markers were better correlated with asymptomatic parasitaemia detected by PCR (r^2^ > 0.29) than with asymptomatic parasitaemia detected by microscopy (r^2^ < 0.13). Asymptomatic parasitaemia detected by microscopy was correlated with asymptomatic parasitaemia detected by PCR (r^2^ = 0.26) and serological markers correlated with each other (r^2^ = 0.40). However, in 2013, when the transmission intensity declined further, the only statistically significant correlations observed were between asymptomatic parasitaemia detected by microscopy and positive PCR tests (r^2^ = 0.11), and between antibodies to MSP1-19 and *Anopheles* mosquitoes (r^2^ = 0.34) (Table 3).

**Table 2:** Correlations between malaria transmission markers in 2012 at 0.9 km resolution

	Clinical malaria cases	Positive blood films	Positive PCR tests	Anti-AMA1 antibody titres	Anti-MSP1-19 antibody titres	*Anopheles* mosquitoes captured
Clinical malaria cases	1					
	266					
Positive blood films	*0.2672*	1				
	*266*	292				
	*<0.0001*					
Positive PCR tests	0.0328	*0.5114*	1			
	266	*292*	292			
	0.5944	*<0.0001*				
Anti-AMA1 antibody titres	0.0215	0.2927	*0.5413*	1		
	41	42	*42*	43		
	0.8937	0.06	*0.0002*			
Anti-MSP1-19 antibody titres	−0.0444	*0.3509*	*0.5745*	*0.6338*	1	
	41	*42*	*42*	*43*	43	
	0.7828	*0.0227*	*0.0001*	*<0.0001*		
*Anopheles* mosquitoes captured	0.0929		0.0648	−0.2398	−0.0218	1
	87	87	87	20	20	90
	0.3923		0.5513	0.3086	0.9272	

All markers are expressed as average per cell of a 0.9 km resolution grid superimposed on the study area. Positive blood films, positive PCR tests and *Anopheles* mosquitoes captured are expressed as mean/grid cell. Antibody titres are expressed as weighted geometric mean/grid cell. Clinical malaria cases are expressed as weighted mean/grid cell. For each pair of markers the table reports from top to bottom the correlation coefficient (r_s_), the number of grid cells (n) and the p value for r_s_

**Table 3:** Correlations between malaria transmission markers in 2013 at 0.9 km resolution

	Clinical malaria cases	Positive blood films	Positive PCR tests	Anti-AMA1 antibody titres	Anti-MSP1-19 antibody titres	*Anopheles* mosquitoes captured
Clinical malaria cases	1					
	266					
Positive blood films	−0.0165	1				
	266	292				
	0.7884					
Positive PCR tests	0.057	*0.3295*	1			
	266	*292*	292			
	0.3544	*<0.0001*				
Anti-AMA1 antibody titres	−0.0339	0.0516	0.002	1		
	22	22	22	22		
	0.8808	0.8196	0.9931			
Anti-MSP1-19 antibody titres	−0.2376	0.0172	−0.0773	−0.0627	1	
	22	22	22	22	22	
	0.2869	0.9394	0.7323	0.7817		
*Anopheles* mosquitoes captured	0.1693	−0.0343	0.116	0.1943	*0.583*	1
	87	87	87	12	*12*	90
	0.1169	0.7527	0.2846	0.5451	*0.0467*	

All markers are expressed as average per cell of a 0.9 km resolution grid superimposed on the study area. Positive blood films, positive PCR tests and *Anopheles* mosquitoes captured are expressed as mean/grid cell. Antibody titres are expressed as weighted geometric mean/grid cell. Clinical malaria cases are expressed as weighted mean/grid cell. For each pair of markers the table reports from top to bottom the correlation coefficient (r_s_), the number of grid cells (n) and the p value for r_s_

## Discussion

The present study describes the fine-scale spatial distribution of *P. falciparum* malaria, examining the relationships between different markers of malaria transmission in an area on the coast of Kenya. The levels of malaria transmission markers seen in this study were lower in 2013 compared with 2012. The low number of asymptomatic infections detected by microscopy prevented us having enough power to detect hotspots in 2013, but we could still detect hotspots with the larger number of positives seen by PCR. Most of the hotspots were unstable (i.e. inconsistent in location between 2012 and 2013), but one hotspot of clinical malaria was maintained in its position and size over the 2 years. When the transmission intensity declined, the spatial correlations observed between the markers were reduced to correlations between parasitological markers on the one hand, and serological and entomological markers on the other hand.

The decline of malaria transmission observed in the present study seems opposite to the observed trends of *P. falciparum* parasite rates along the coast [29]. However, this study was conducted in a restricted area and previous studies have reported high rates of heterogeneity in malaria transmission in the region, and that differing trends can be observed in sub-locations within the same area [3].

From 2012 to 2013, 57 % of hotspots disappeared and among the remaining ones, only PCR and serology hotspots maintained a size above the homestead level; the other hotspots shrank to single homesteads. Hotspots of asymptomatic parasite carriers detected by microscopy were not seen in 2013 despite ongoing transmission evidenced by clinical malaria cases. This suggests that cross-sectional surveys using microscopy may not be ideal when transmission intensity declines to very low levels [30]. The hotspots that persisted were asymptomatic parasite carriers detected by PCR and serological methods, suggesting these markers as good candidates for sensitive hotspot detection in settings with declining malaria transmission.

In 2012, there was a total overlap of hotspots of asymptomatic parasite carriers detected by microscopy with hotspots of asymptomatic parasite carriers detected by PCR as expected, especially given that the sensitivity of PCR is higher than that of light microscopy [31]. This is consistent with the statistically significant spatial correlation observed between these markers when the data were analysed at grid cell level. On the other hand, epidemiological studies have shown that parasite density can be inversely proportional to the intensity of transmission at a micro-epidemiological scale [32], and this might have led microscopy-defined hotspots to be differently located from PCR-defined hotspots. In the present study the transmission intensity was much lower and this phenomenon was not observed. The hotspots of asymptomatic parasite carriers detected by PCR overlapped the hotspots detected by serological markers in 2012 and 2013, supporting the idea of using serological markers as an alternative to PCR in the detection of hotspots. It has been shown previously that children living in hotspots of asymptomatic parasitaemia have higher antibody titres compared with those living in clinical malaria hotspots [2], and antibody titres have been described as a marker of exposure [33].

One clinical malaria hotspot was found to be stable across the 2 years, which is not consistent with previous reports in which hotspots of clinical malaria were found to be unstable compared with hotspots of asymptomatic parasitaemia [2]. However, the short period of observation in the present study and the low age of children assessed (i.e. 5–17 months olds) in a low transmission setting are the likely explanation of this observation since immunity to clinical malaria builds up over a much longer period at lower transmission intensities [34, 35].

The relative simplicity and lower cost of serology compared with PCR may make the use of serological markers more attractive for large-scale surveillance. However the fact that serological surveys may not distinguish recent from medium-term exposure may be a disadvantage, since the location of hotspots may vary from year to year. This could be overcome by including only young children in the surveys [36] whose antibody responses have been attributed to short-lived plasma cells [37, 38] or by measuring responses to antigens for which evidence suggest that they have limited capacity to induce long-lived plasma cells [39]. Helb et al. have recently reported that antibody responses to some novel malaria antigens can limit detection to infections occurring within the last 30 days [40].

The present study was opportunistic based on the availability of datasets and thus presents with some limitations. The sampled homesteads were not the same for the entomological, serological and clinical surveillance studies and this prevents us from examining homestead-level correlations in more detail. The sample size was not large and the limited period of observation does not allow a definitive assessment of temporal stability.

## Conclusions

The global decline of malaria transmission and plans for elimination have led to increased interest in the fine-scale epidemiology of malaria. One of the challenges in targeted interventions is the appropriate detection of residual transmission foci at the pre-elimination stage. The choice of a cost-effective marker that can be logistically feasible and readily implemented across sites by malaria control programmes would be important in the elimination efforts as well as the post-elimination surveillance. These findings may support the choice of either serology or PCR as markers in the detection of transmission hotspots for targeted interventions.

### Availability of supporting data

The supporting data are under the custodianship of the KEMRI-Wellcome Trust Data Governance Committee and is accessible upon request addressed to that committee.

## Additional files

10.1186/s12936-016-1260-3 Distribution of study population by age groups in the cross-sectional surveys.
10.1186/s12936-016-1260-3 Tessellation of the study area and densities of homesteads. Each grid cell is a 0.9 × 0.9 km square. In figure A the green dots represent the homesteads. In figure B the shades of green color are proportional to the densities of homesteads with darker shades representing higher densities.
10.1186/s12936-016-1260-3 Dynamics of hotspots of asymptomatic parasite carriers detected by PCR.
10.1186/s12936-016-1260-3 Overlapping of malaria transmission hotspots detected in 2012 with those detected in 2013. Empty cells indicates no intersection and no overlapping was observed i.e. *d* > *R* + *r*. A ratio = 1 indicates a total overlapping i.e. *d* = 0 and *R* = *r*, or *d* < *R* -* r*, where *d* is the distance between the centres of two given hotspots, *R* and* r* the radii of these hotspots. *HS index* hotspot index in relation to Additional file 8.
10.1186/s12936-016-1260-3 Spatial overlapping of hotspots of serological markers of malaria transmission. Each green dot represents a homestead. The homesteads sampled in 2012 differed from those sampled in 2013.
10.1186/s12936-016-1260-3 Spatial overlapping of hotspots of clinical and parasitological markers of malaria transmission. The homesteads sampled in 2012 were the same as those sampled in 2013 for the longitudinal study.
10.1186/s12936-016-1260-3 Overlapping of malaria transmission hotspots in 2012. Empty cells indicate no intersection and no overlapping was observed i.e. *d* > *R* + *r*. A ratio = 1 indicates a total overlapping i.e. *d* = 0 and *R* = *r*, or *d* < *R* - *r*, where *d* is the distance between the centres of two given hotspots, *R* and *r* the radii of these hotspots. *HS index* hotspot index in relation to Additional file 8. *NA* not applicable.
10.1186/s12936-016-1260-3 Summary of the spatial overlapping of hotspots of malaria transmission markers. Each green spot represents a homestead. All homesteads involved in the clinical surveillance, serology and entomology studies and the specific hotspots are superimposed on the same map for each year.
10.1186/s12936-016-1260-3 Overlapping of malaria transmission hotspots in 2013. Empty cells indicate no intersection and no overlapping was observed i.e. *d* > *R* + *r*. A ratio = 1 indicates a total overlapping i.e. *d* = 0 and *R* = *r*, or *d* < *R* - *r*, where *d* is the distance between the centres of two given hotspots, *R* and *r* the radii of these hotspots. *HS index* hotspot index in relation to Additional file 8. *NA* not applicable.
10.1186/s12936-016-1260-3 Distribution of antibody titres to AMA1 and MSP1-19. The data are aggregated at homestead level.
10.1186/s12936-016-1260-3 Distribution of clinical, parasitological and entomological markers of malaria transmission. The data are aggregated at homestead level.
10.1186/s12936-016-1260-3 Correlations between clinical, parasitological, serological and entomological malaria transmission markers. All markers are expressed as average per cell of a 0.9 km resolution grid superimposed on the study area. Positive blood films, positive PCR tests and *Anopheles* mosquitoes captured are expressed as mean/grid cell. Antibody titres are expressed as weighted geometric mean/grid cell. Clinical malaria cases are expressed as weighted mean/grid cell.

## Abbreviations

AMA1: apical membrane antigen 1
CDC: Centers for Disease Control and Prevention
ELISA: enzyme-linked immunosorbent assay
KEMRI: Kenya Medical Research Institute
MSP1-19: merozoite surface protein 1
PCR: polymerase chain reaction
RR: relative risk

## References

[1] Carter R, Mendis KN (2002). Evolutionary and historical aspects of the burden of malaria. Clin Microbiol Rev.

[2] Bejon P, Williams TN, Liljander A, Noor AM, Wambua J, Ogada E (2010). Stable and unstable malaria hotspots in longitudinal cohort studies in Kenya. PLoS Med.

[3] Bejon P, Williams TN, Nyundo C, Hay SI, Benz D, Gething PW (2014). A micro-epidemiological analysis of febrile malaria in Coastal Kenya showing hotspots within hotspots. ELife.

[4] Midega JT, Smith DL, Olotu A, Mwangangi JM, Nzovu JG, Wambua J (2012). Wind direction and proximity to larval sites determines malaria risk in Kilifi District in Kenya. Nat Commun.

[5] Brooker S, Clarke S, Njagi JK, Polack S, Mugo B, Estambale B (2004). Spatial clustering of malaria and associated risk factors during an epidemic in a highland area of western Kenya. Trop Med Int Health.

[6] De Silva PM, Marshall JM (2012). Factors contributing to urban malaria transmission in sub-Saharan Africa: a systematic review. J Trop Med.

[7] Tusting L, Ippolito M, Willey B, Kleinschmidt I, Dorsey G, Gosling R (2015). The evidence for improving housing to reduce malaria: a systematic review and meta-analysis. Malar J.

[8] Amoako N, Asante KP, Adjei G, Awandare GA, Bimi L, Owusu-Agyei S (2014). Associations between red cell polymorphisms and *Plasmodium falciparum* infection in the middle belt of Ghana. PLoS One.

[9] Sonko ST, Jaiteh M, Jafali J, Jarju LBS, D’Alessandro U, Camara A (2014). Does socio-economic status explain the differentials in malaria parasite prevalence? Evidence from The Gambia. Malar J.

[10] Yadouléton A, N’Guessan R, Allagbé H, Asidi A, Boko M, Osse R (2010). The impact of the expansion of urban vegetable farming on malaria transmission in major cities of Benin. Parasit Vectors.

[11] Fernández-Grandon GM, Gezan SA, Armour JAL, Pickett JA, Logan JG (2015). Heritability of attractiveness to mosquitoes. PLoS One.

[12] Lacroix R, Mukabana WR, Gouagna LC, Koella JC (2005). Malaria infection increases attractiveness of humans to mosquitoes. PLoS Biol.

[13] Kicska GA, Ting LM, Schramm VL, Kim K (2003). Effect of dietary p-aminobenzoic acid on murine *Plasmodium yoelii* infection. J Infect Dis.

[14] Pasvol G, Weatherall DJ, Wilson RJM, Smith DH, Gilles HM (1976). Fetal haemoglobin and malaria. Lancet.

[15] Malaria Genomic Epidemiology Network (2015). A novel locus of resistance to severe malaria in a region of ancient balancing selection. Nature.

[16] Bousema T, Griffin JT, Sauerwein RW, Smith DL, Churcher TS, Takken W (2012). Hitting hotspots: spatial targeting of malaria for control and elimination. PLoS Med.

[17] Mosha J, Sturrock H, Greenwood B, Sutherland C, Gadalla N, Atwal S (2014). Hot spot or not: a comparison of spatial statistical methods to predict prospective malaria infections. Malar J.

[18] Bousema T, Drakeley C, Gesase S, Hashim R, Magesa S, Mosha F (2010). Identification of hot spots of malaria transmission for targeted malaria control. J Infect Dis.

[19] Nourein AB, Abass MA, Nugud AHD, El Hassan I, Snow RW, Noor AM (2011). Identifying residual foci of *Plasmodium falciparum* infections for malaria elimination: the urban context of Khartoum, Sudan. PLoS One.

[20] Mirghani S, Nour B, Bushra S, Elhassan I, Snow R, Noor A (2010). The spatial-temporal clustering of *Plasmodium falciparum* infection over eleven years in Gezira State, The Sudan. Malar J.

[21] RTS,S Clinical Trials Partnership (2015). Efficacy and safety of RTS,S/AS01 malaria vaccine with or without a booster dose in infants and children in Africa: final results of a phase 3, individually randomised, controlled trial. Lancet.

[22] Mwangi TW, Ross A, Snow RW, Marsh K (2005). Case definitions of clinical malaria under different transmission conditions in Kilifi District, Kenya. J Infect Dis.

[23] Andrews L, Andersen RF, Webster D, Dunachie S, Walther RM, Bejon P (2005). Quantitative real-time polymerase chain reaction for malaria diagnosis and its use in malaria vaccine clinical trials. Am J Trop Med Hyg.

[24] Stewart L, Gosling R, Griffin J, Gesase S, Campo J, Hashim R (2009). Rapid assessment of malaria transmission using age-specific sero-conversion rates. PLoS One.

[25] Kulldorff M (1997). A spatial scan statistic. Commun Stat Theor Methods.

[26] Bousema T, Youssef RM, Cook J, Cox J, Alegana VA, Amran J (2010). Serologic markers for detecting malaria in areas of low endemicity, Somalia, 2008. Emerg Infect Dis.

[27] Weisstein EW. Circle-circle intersection. From *MathWorld*-A Wolfram web resource. 2016. http://www.mathworld.wolfram.com/Circle-CircleIntersection.html. Accessed 02 Mar 2016.

[28] WRI. Kenya GIS Data. 2007. http://www.wri.org/resources/data-sets/kenya-gis-data. Accessed 04 Aug 2015.

[29] Snow RW, Kibuchi E, Karuri SW, Sang G, Gitonga CW, Mwandawiro C (2015). Changing malaria prevalence on the Kenyan coast since 1974: climate, drugs and vector control. PLoS One.

[30] Bousema T, Okell L, Felger I, Drakeley C (2014). Asymptomatic malaria infections: detectability, transmissibility and public health relevance. Nat Rev Microbiol.

[31] Bejon P, Andrews L, Hunt-Cooke A, Sanderson F, Gilbert S, Hill A (2006). Thick blood film examination for *Plasmodium falciparum* malaria has reduced sensitivity and underestimates parasite density. Malar J.

[32] Mosha JF, Sturrock HJW, Greenhouse B, Greenwood B, Sutherland CJ, Gadalla N (2013). Epidemiology of subpatent *Plasmodium falciparum* infection: implications for detection of hotspots with imperfect diagnostics. Malar J.

[33] Badu K, Gyan B, Appawu M, Mensah D, Dodoo D, Yan G (2015). Serological evidence of vector and parasite exposure in Southern Ghana: the dynamics of malaria transmission intensity. Parasit Vectors.

[34] Griffin JT, Hollingsworth TD, Reyburn H, Drakeley CJ, Riley EM, Ghani AC (2015). Gradual acquisition of immunity to severe malaria with increasing exposure. Proc Biol Sci.

[35] Langhorne J, Ndungu FM, Sponaas A-M, Marsh K (2008). Immunity to malaria: more questions than answers. Nat Immunol.

[36] Singer LM, Mirel LB, ter Kuile FO, Branch OH, Vulule JM, Kolczak MS (2003). The effects of varying exposure to malaria transmission on development of antimalarial antibody responses in preschool children. XVI. Asembo Bay Cohort Project. J Infect Dis.

[37] Weiss GE, Traore B, Kayentao K, Ongoiba A, Doumbo S, Doumtabe D (2010). The *Plasmodium falciparum*-specific human memory b cell compartment expands gradually with repeated malaria infections. PLoS Pathog.

[38] Kinyanjui SM, Conway DJ, Lanar DE, Marsh K (2007). IgG antibody responses to *Plasmodium falciparum* merozoite antigens in Kenyan children have a short half-life. Malar J.

[39] Proietti C, Verra F, Bretscher MT, Stone W, Kanoi BN, Balikagala B (2013). Influence of infection on malaria-specific antibody dynamics in a cohort exposed to intense malaria transmission in northern Uganda. Parasite Immunol.

[40] Helb DA, Tetteh KKA, Felgner PL, Skinner J, Hubbard A, Arinaitwe E (2015). Novel serologic biomarkers provide accurate estimates of recent *Plasmodium falciparum* exposure for individuals and communities. Proc Natl Acad Sci USA.

