# Supplementary material for: Malaria hotspots defined by clinical malaria, asymptomatic carriage, PCR and vector numbers in a low transmission area on the Kenyan Coast
Source: Malar J. 2016 Apr 14;15:213. doi: 10.1186/s12936-016-1260-3 (PMC4831169; doi:10.1186/s12936-016-1260-3)
Supplement: Supplementary file 1 — 10.1186/s12936-016-1260-3 Distribution of study population by age groups in the cross-sectional surveys. [file 12936_2016_1260_MOESM1_ESM.docx]

| Age groups | Numbers |
| --- | --- |
| 6 months – 5 years | 400 |
| - 1. ears | 200 |
| > 20 years | 200 |
| Total | 800 |
