# Supplementary figures and images for: Malaria hotspots defined by clinical malaria, asymptomatic carriage, PCR and vector numbers in a low transmission area on the Kenyan Coast
Source: Malar J. 2016 Apr 14;15:213. doi: 10.1186/s12936-016-1260-3 (PMC4831169; doi:10.1186/s12936-016-1260-3)

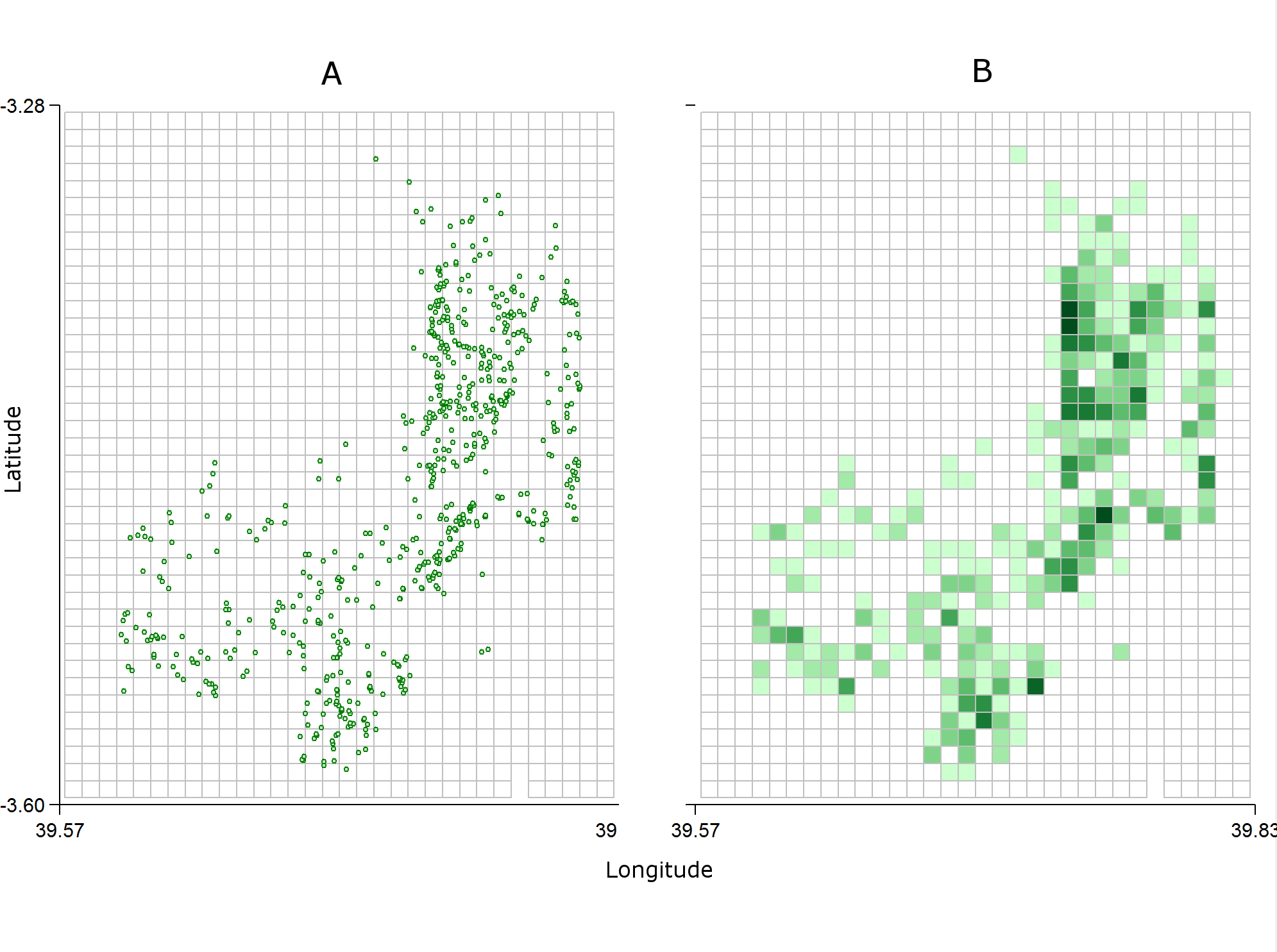

Supplement: Supplementary file 2 — 10.1186/s12936-016-1260-3 Tessellation of the study area and densities of homesteads. Each grid cell is a 0.9 × 0.9 km square. In figure A the green dots represent the homesteads. In figure B the shades of green color are proportional to the densities of homesteads with darker shades representing higher densities. [file 12936_2016_1260_MOESM2_ESM.tif]

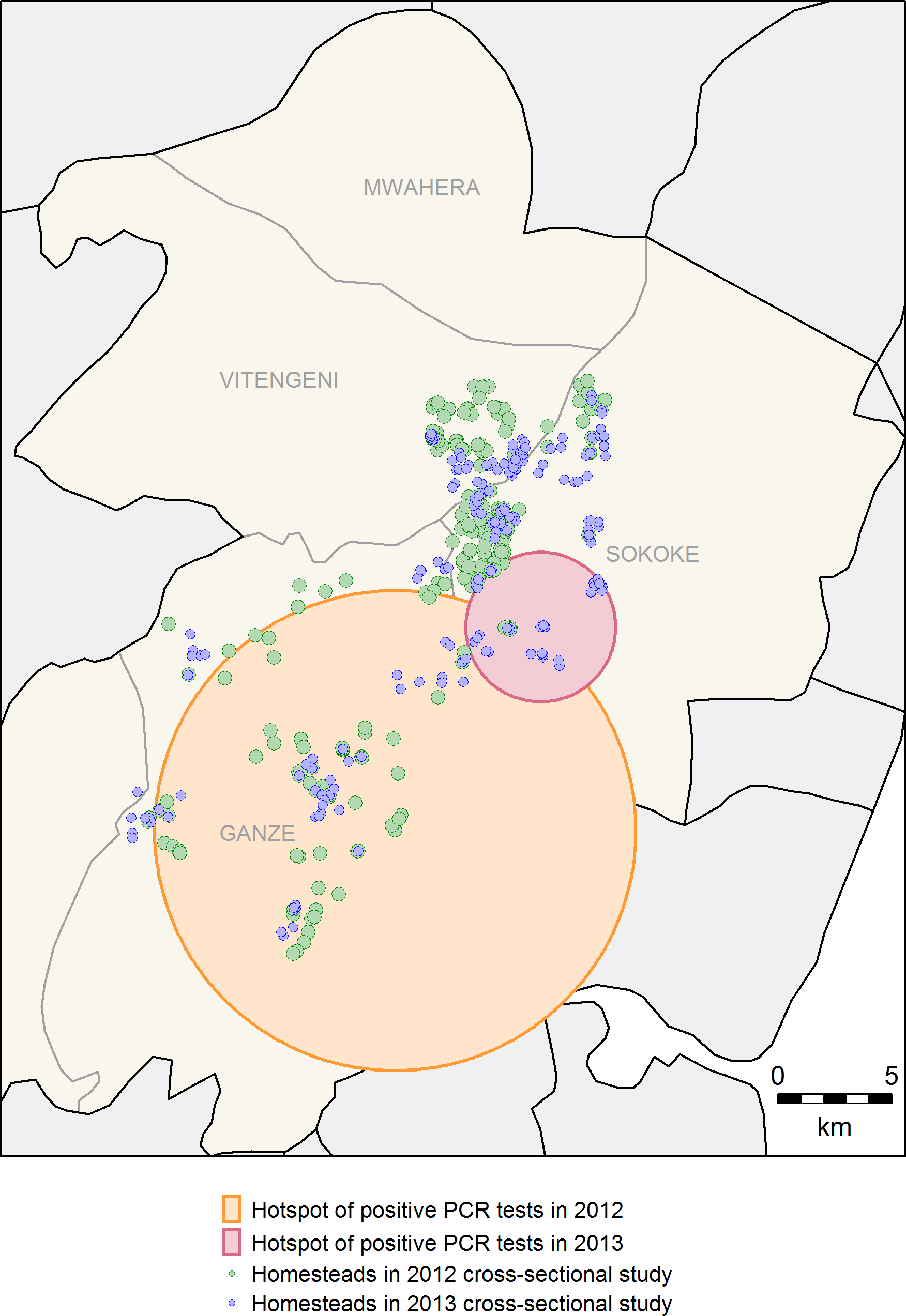

Supplement: Supplementary file 3 — 10.1186/s12936-016-1260-3 Dynamics of hotspots of asymptomatic parasite carriers detected by PCR. [file 12936_2016_1260_MOESM3_ESM.tif]

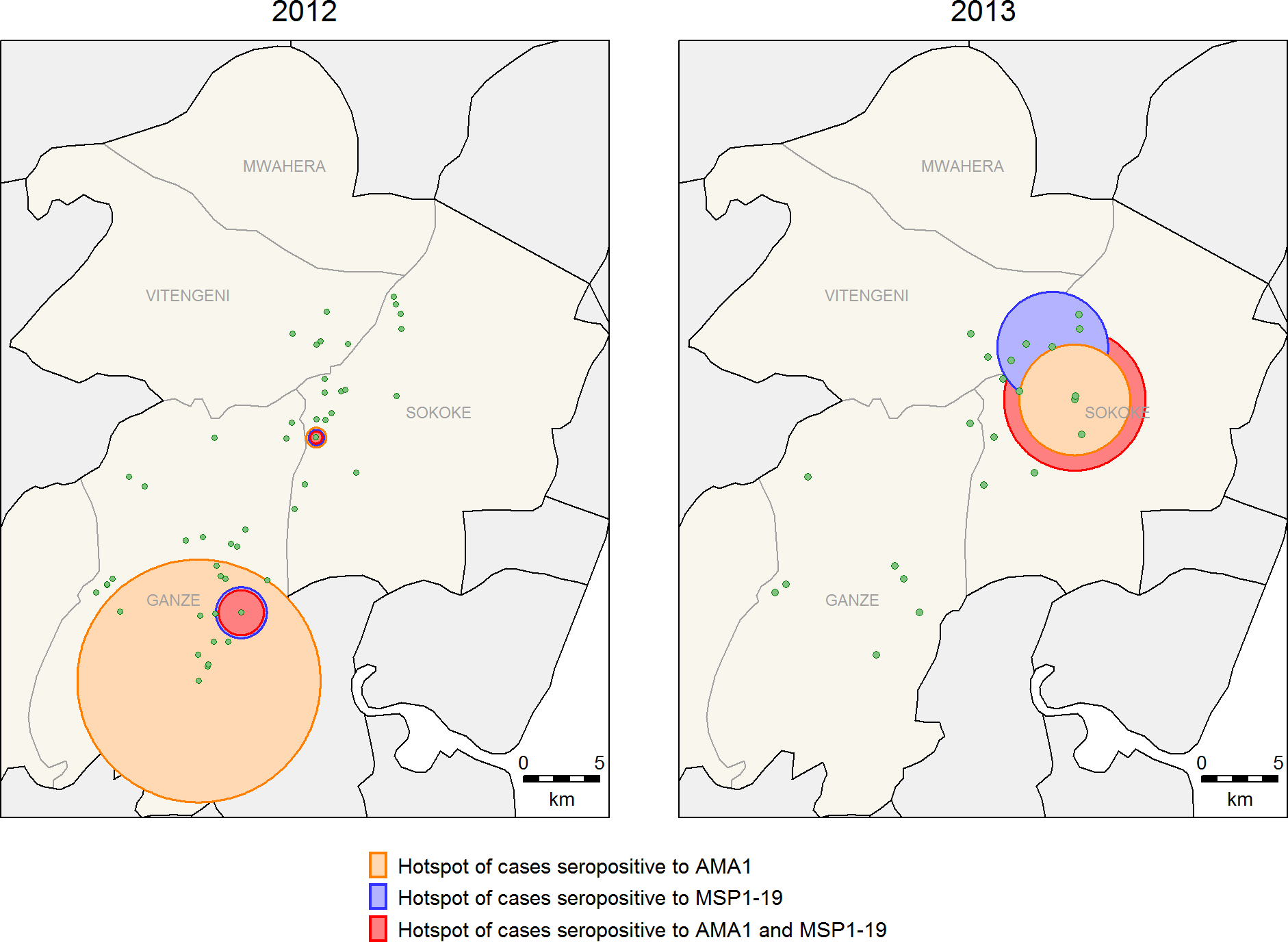

Supplement: Supplementary file 5 — 10.1186/s12936-016-1260-3 Spatial overlapping of hotspots of serological markers of malaria transmission. Each green dot represents a homestead. The homesteads sampled in 2012 differed from those sampled in 2013. [file 12936_2016_1260_MOESM5_ESM.tif]

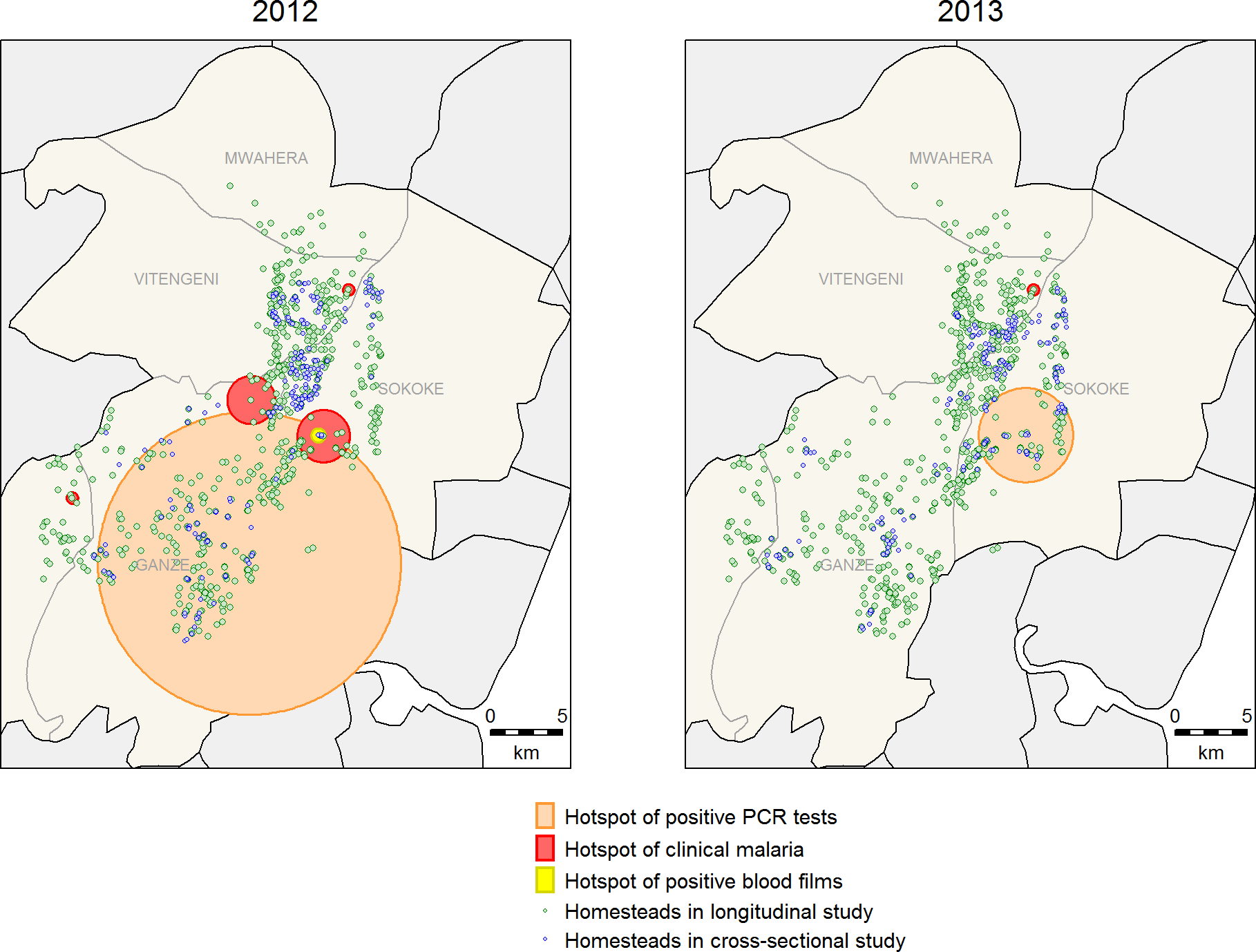

Supplement: Supplementary file 6 — 10.1186/s12936-016-1260-3 Spatial overlapping of hotspots of clinical and parasitological markers of malaria transmission. The homesteads sampled in 2012 were the same as those sampled in 2013 for the longitudinal study. [file 12936_2016_1260_MOESM6_ESM.tif]

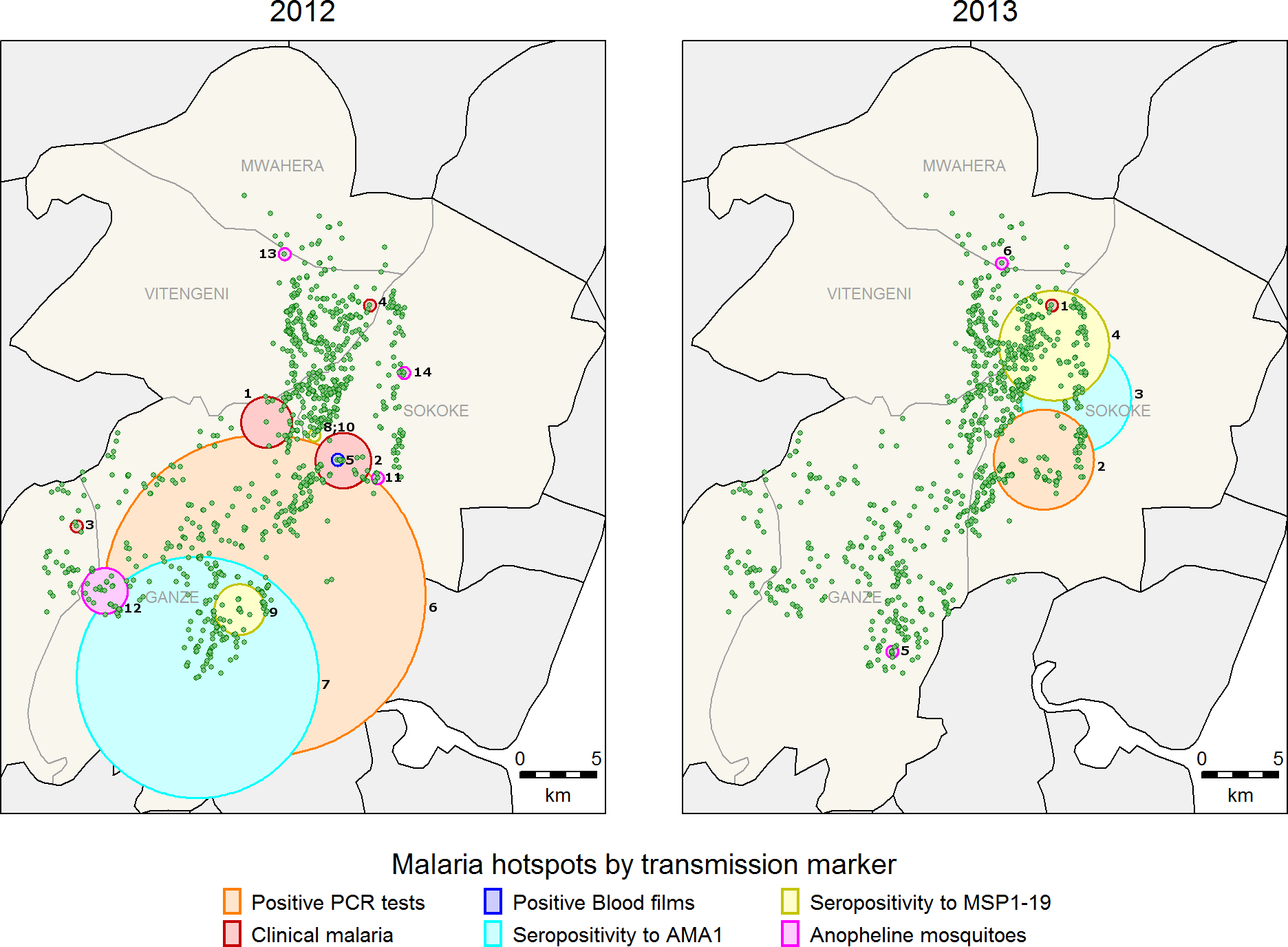

Supplement: Supplementary file 8 — 10.1186/s12936-016-1260-3 Summary of the spatial overlapping of hotspots of malaria transmission markers. Each green spot represents a homestead. All homesteads involved in the clinical surveillance, serology and entomology studies and the specific hotspots are superimposed on the same map for each year. [file 12936_2016_1260_MOESM8_ESM.tif]

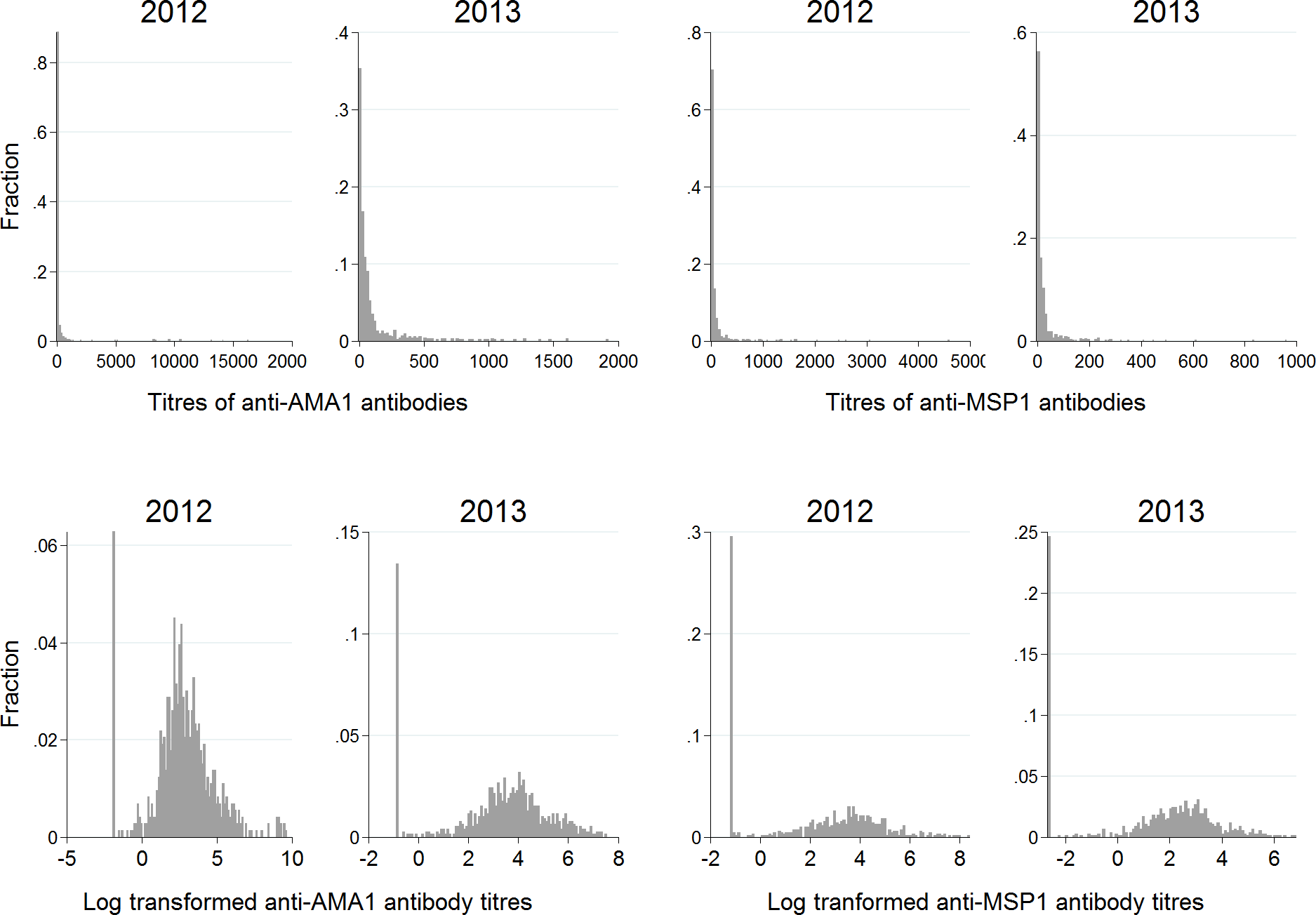

Supplement: Supplementary file 10 — 10.1186/s12936-016-1260-3 Distribution of antibody titres to AMA1 and MSP1-19. The data are aggregated at homestead level. [file 12936_2016_1260_MOESM10_ESM.tif]

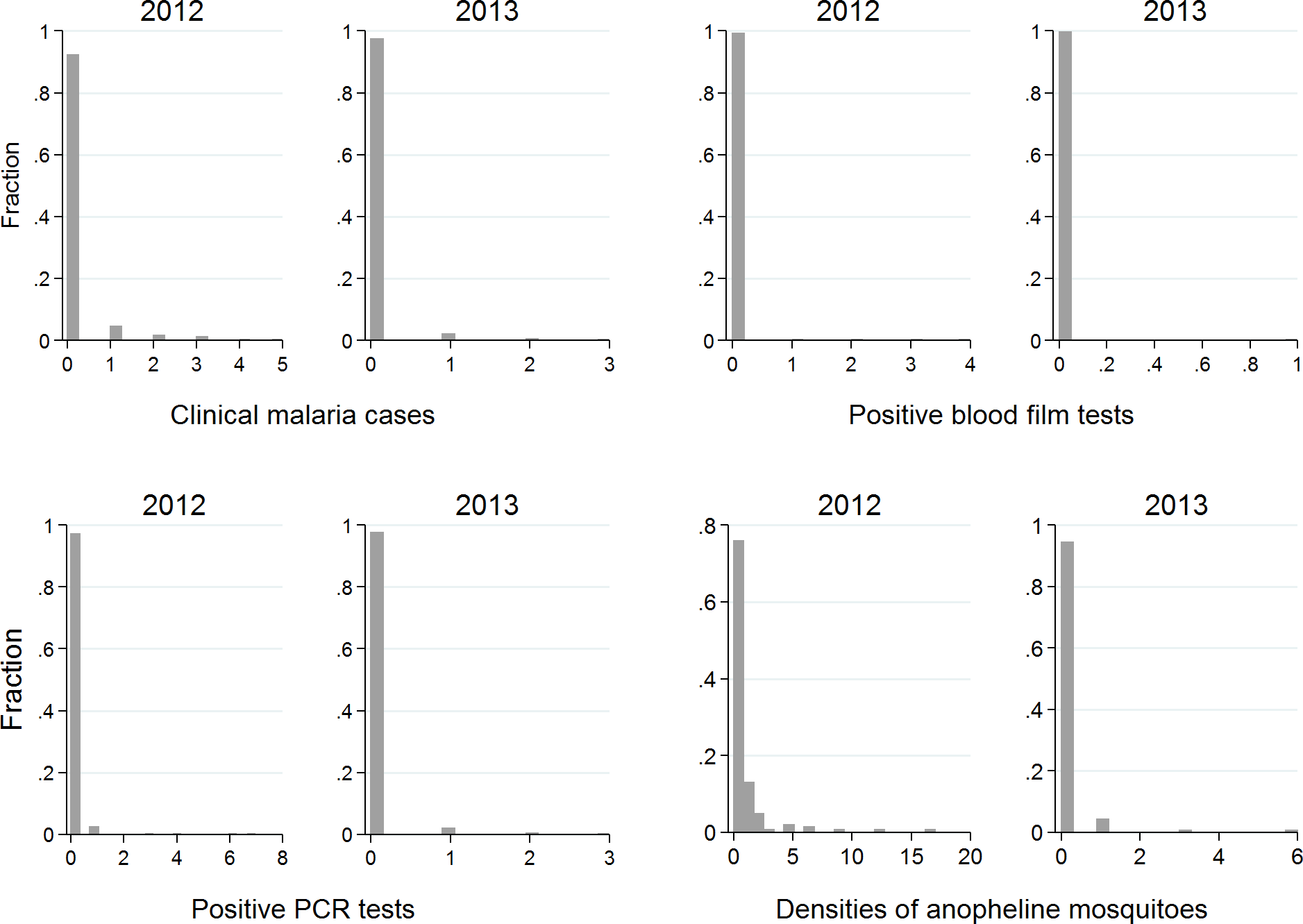

Supplement: Supplementary file 11 — 10.1186/s12936-016-1260-3 Distribution of clinical, parasitological and entomological markers of malaria transmission. The data are aggregated at homestead level. [file 12936_2016_1260_MOESM11_ESM.tif]

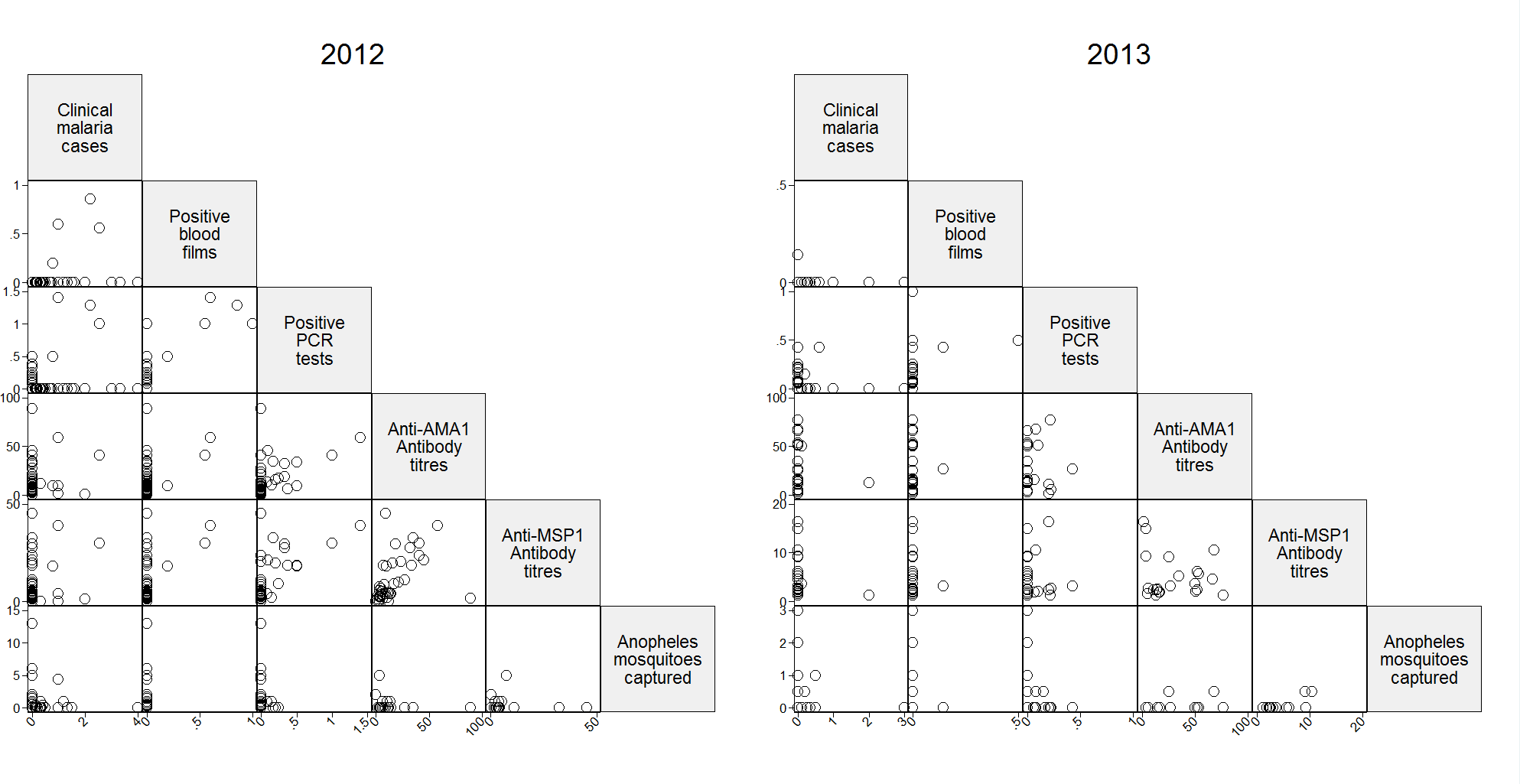

Supplement: Supplementary file 12 — 10.1186/s12936-016-1260-3 Correlations between clinical, parasitological, serological and entomological malaria transmission markers. All markers are expressed as average per cell of a 0.9 km resolution grid superimposed on the study area. Positive blood films, positive PCR tests and Anopheles mosquitoes captured are expressed as mean/grid cell. Antibody titres are expressed as weighted geometric mean/grid cell. Clinical malaria cases are expressed as weighted mean/grid cell. [file 12936_2016_1260_MOESM12_ESM.tif]
