# Supplementary material for: Malaria hotspots defined by clinical malaria, asymptomatic carriage, PCR and vector numbers in a low transmission area on the Kenyan Coast
Source: Malar J. 2016 Apr 14;15:213. doi: 10.1186/s12936-016-1260-3 (PMC4831169; doi:10.1186/s12936-016-1260-3)
Supplement: Supplementary file 4 — 10.1186/s12936-016-1260-3 Overlapping of malaria transmission hotspots detected in 2012 with those detected in 2013. Empty cells indicates no intersection and no overlapping was observed i.e. d > R + r. A ratio = 1 indicates a total overlapping i.e. d = 0 and R = r, or d < R - r, where d is the distance between the centres of two given hotspots, R and r the radii of these hotspots. HS index hotspot index in relation to Additional file 8. [file 12936_2016_1260_MOESM4_ESM.docx]

| Year |  | 2013 | | | | | | |
| --- | --- | --- | --- | --- | --- | --- | --- | --- |
|  | Marker |  | Clinical malaria | PCR | Seropositive  AMA1 | Seropositive  MSP1 | Anopheles mosquitoes | |
| 2012 |  | HS index | 1 | 2 | 3 | 4 | 5 | 6 |
|  | Clinical malaria | 1 | - | - | - | - | - | - |
|  |  | 2 | - | 1 | 0.011 | - | - | - |
|  |  | 3 | - | - | - | - | - | - |
|  |  | 4 | 1 | - | - | 1 | - | - |
|  | Microscopy | 5 | - | 1 | - | - | - | - |
|  | PCR | 6 | **-** | 0.39 | - | - | 1 | - |
|  | Seropositive AMA1 | 7 | - | **-** | - | - | 1 | - |
|  |  | 8 | - | - | - | - | - | - |
|  | Seropositive MSP1 | 9 | - | **-** | **-** | - | - | - |
|  |  | 10 | - | - | - | - | - | - |
|  | Anopheles mosquitoes | 11 | - | 1 | - | - | - | - |
|  |  | 12 | - | **-** | **-** | - | - | - |
|  |  | 13 | - | - | - | **-** | - | - |
|  |  | 14 | - | - | 1 | 1 | - | - |
