# Supplementary material for: Malaria hotspots defined by clinical malaria, asymptomatic carriage, PCR and vector numbers in a low transmission area on the Kenyan Coast
Source: Malar J. 2016 Apr 14;15:213. doi: 10.1186/s12936-016-1260-3 (PMC4831169; doi:10.1186/s12936-016-1260-3)
Supplement: Supplementary file 9 — 10.1186/s12936-016-1260-3 Overlapping of malaria transmission hotspots in 2013. Empty cells indicate no intersection and no overlapping was observed i.e. d > R + r. A ratio = 1 indicates a total overlapping i.e. d = 0 and R = r, or d < R - r, where d is the distance between the centres of two given hotspots, R and r the radii of these hotspots. HS index hotspot index in relation to Additional file 8. NA not applicable. [file 12936_2016_1260_MOESM9_ESM.docx]

| Marker |  | Clinical  malaria | PCR | Seropositive  AMA1 | Seropositive  MSP1 | Anopheles  mosquitoes | |
| --- | --- | --- | --- | --- | --- | --- | --- |
|  | HS index | 1 | 2 | 3 | 4 | 5 | 6 |
| Clinical malaria | 1 | NA |  |  |  |  |  |
| PCR | 2 | - | NA |  |  |  |  |
| Seropositive AMA1 | 3 | - | **0.25** | NA |  |  |  |
| Seropositive MSP1 | 4 | **1** | - | **0.38** | NA |  |  |
| Anopheles  mosquitoes | 5 | - | - | - | - | NA |  |
|  | 6 | - | - | - | - | - | NA |
